# Leisure Engagement and Self-Perceptions of Aging: Longitudinal Analysis of Concurrent and Lagged Relationships

**DOI:** 10.1093/geronb/gbad182

**Published:** 2023-12-22

**Authors:** Feifei Bu, Hei Wan Mak, Jessica K Bone, Qian Gao, Jill K Sonke, Daisy Fancourt

**Affiliations:** Research Department of Behavioural Science and Health, Institute of Epidemiology and Health Care, University College London, London, UK; Research Department of Behavioural Science and Health, Institute of Epidemiology and Health Care, University College London, London, UK; Research Department of Behavioural Science and Health, Institute of Epidemiology and Health Care, University College London, London, UK; Research Department of Behavioural Science and Health, Institute of Epidemiology and Health Care, University College London, London, UK; Center for Arts in Medicine, University of Florida, Gainesville, Florida, USA; Research Department of Behavioural Science and Health, Institute of Epidemiology and Health Care, University College London, London, UK; (Psychological Sciences Section)

**Keywords:** Aging satisfaction, Arts, Cultural engagement, Healthy aging, Physical activity

## Abstract

**Objectives:**

There is evidence to suggest that leisure engagement may influence self-perceptions of aging, but disentangling potential bidirectionality in this relationship is challenging. A better understanding of the directionality of this association is essential for designing more effective interventions to promote healthy aging. We, therefore, tested concurrent effects and lagged effects in both directions for a univariate measure of leisure engagement as well as specific domains of community, cognitive, creative, and physical activities.

**Methods:**

A total of 17,753 adults aged 50 or above living in the United States from the Health and Retirement Study were included in the analysis. They provided 32,703 observations over 3 waves between 2008/2010 and 2016/2018. Data were analyzed using structural equation modeling with both concurrent and lagged associations between self-perceptions of aging and leisure engagement, controlling for confounders including age, gender, ethnicity, socioeconomic position, and health conditions.

**Results:**

We found consistent evidence for leisure engagement as a predictor of self-perceptions of aging. There was also evidence for a reciprocal relationship where leisure engagement was predicted by older adults’ self-perceptions of aging. Similar results were observed for specific domains of leisure engagement.

**Discussion:**

Our findings provide empirical support for the potential benefits of leisure engagement on positive self-perceptions of aging, regardless of the type of activities. Our study also highlights the importance to consider the directionality in researching leisure engagement and self-perceptions of aging.

In recent years, there has been an increased interest in self-perceptions of aging (SPA), which can be defined as people’s perceptions of themselves and their aging process when they become older (Levy, 2003). SPA is recognized as a multidimensional construct rooted in both positive and negative personal aging experiences. It goes beyond the simple constructs of subjective age and age identity, all of which can be seen as components of a superordinate construct of awareness of aging (Diehl et al., 2014). Positive SPA is an important indicator and promoter of healthy and successful aging. Empirical studies have shown that SPA is related to both physical and mental functioning among older adults, including mortality/longevity (Christensen et al., 2009; Kotter-Grühn et al., 2009; Westerhof et al., 2014), functional health (Levy et al., 2002; Moser et al., 2011; Tovel et al., 2019), cognitive function (Robertson et al., 2015), psychological well-being (Freeman et al., 2016; Nakamura et al., 2022), health behaviors (Levy & Myers, 2004; Nakamura et al., 2022), and the use of preventive health services (Kim et al., 2014).

There are many factors that can shape SPA, including individual characteristics, expectations, and experiences in different life domains, as well as societal–cultural level contexts (Diehl et al., 2015). More relevant to the present study, leisure engagement such as physical activity, gardening, volunteering, arts and cultural activities, community, and other activities that people engage in during their free time may influence how they evaluate their own aging process. Leisure engagement often involves components that comprise sensory, cognitive, or creative stimuli and physical bodily motions or actions (Warran et al., 2022), which can trigger positive emotions that influence individuals’ thoughts, behaviors, and well-being (Fancourt et al., 2021). For instance, the arts have been shown to elicit positive affect, increase happiness, help regulate emotions, and cope with adversity; and group participation facilitates interpersonal connections, builds meaningful relationships, evokes greater empathy, develops trusted resources, and encourages prosocial behaviors (Fancourt & Finn, 2019). Physical activity is shown to enhance the sense of self-efficacy and personal control, stimulate neuronal regeneration in the hippocampus, preserve brain volume, and improve a wide range of chronic condition symptoms (Brothers & Diehl, 2017; Stewart, 2005). All of these could help reduce negative aging stereotypes, improve mutual understanding (Ory et al., 2003), enhance positive aging experiences, and thus lead to positive aging perceptions. It is important to note that different leisure activities may have different impacts on SPA, or have similar influences but through different active ingredients or pathways.

However, SPA might also influence leisure engagement (Hicks & Siedlecki, 2017). The stereotype embodiment theory proposes that stereotypes of aging are embodied within social and cultural settings (Levy, 2009). Such stereotypes can become internalized across the life span and may operate unconsciously through three pathways: psychological, behavioral, and physiological (Levy, 2009). Psychologically, age stereotypes may generate expectations for older adults that act as self-fulfilling prophecies, affecting the way they behave in order to “meet” those expectations. For instance, a negative labeling of aging may lead one to build negative SPA, raise more aging-related concerns, feel less capable of engaging in leisure activities, and thus become less inclined to engage in these activities (Hess & Hinson, 2006; Levy et al., 2014). Through the behavioral pathway, older adults with more positive SPA may be more likely to engage in health-promoting behaviors, compared to those who hold more negative SPA. For example, a study in Germany (*n* = 5,194) found that middle-aged and older adults with more positive SPA engaged in a higher frequency of leisure activities (e.g., doing sports, going for walks, board games, engaging in arts and cultural activities), which partially explained the association between SPA and physical health (Hicks & Siedlecki, 2017). Finally, physiologically, older adults with more negative views of their own aging process may have poorer functional health (e.g., Levy et al., 2002), which prevents them from accessing and engaging in leisure activities. There are thus a number of potential causal pathways linking SPA to leisure engagement.

Despite theoretical explanations for both directions, the directionality of the relationship between leisure engagement and SPA remains unclear due to a lack of empirical evidence. A better understanding of the directionality is essential for designing more effective interventions to promote healthy aging. Therefore, this study aimed to examine the relationship between leisure engagement and SPA among older adults using data from the Health and Retirement Study (HRS; Sonnega et al., 2014). We tested the following hypotheses: (1) leisure engagement predicts SPA; (2) SPA also predicts leisure engagement. Although leisure engagement is treated as a univariate construct in most empirical studies, it consists of a diverse range of activities that could have differential benefits (Fancourt et al., 2021), which may be multivariate or multidimensional (VanderWeele, 2022). Therefore, we further hypothesized that (3) different domains of leisure engagement may be differentially associated with SPA.

## Method

### Data

Participants were drawn from the RAND HRS longitudinal file 2018 (V1). HRS is a nationally representative study of more than 37,000 individuals over the age of 50 and their partners in the United States (Sonnega et al., 2014). The initial cohort was first interviewed in 1992 and followed up every 2 years, and the sample is replenished with younger cohorts every 6 years (Sonnega et al., 2014).

Since 2006, HRS has collected psychosocial and lifestyle data in each biennial wave from a rotating random 50% subsample of participants using the Leave-Behind Psychosocial and Lifestyle Questionnaire (LBQ), which included questions on leisure activities and SPA (Smith et al., 2017). Response rates in each year varied from 62% to 85%. We excluded data collected in 2006 where the engagement in leisure activities was measured differently, using the 2008–2018 data. The rotating random subsamples form two subpanels, with one completing the LBQ in 2008, 2012, and 2016, and the other in 2010, 2014, and 2018. To maximize the sample size, we combined the two subpanels into one panel which was followed up every 4 years. There were three waves of longitudinal data at 4-year intervals, with the baseline wave in 2008/2010 (Supplementary Figure 1).

In total, 21,569 participants completed at least one wave of the LBQ, and 20,235 (93.8%) had valid leisure engagement measures. Restricting this sample to participants with at least one wave of data on SPA reduced the sample to 19,291 (89.4%). After excluding participants with missing data on any of the covariates (see later), we were left with an analytical sample of 17,753 participants (82.3%) and 32,703 observations (1.8 repeated measures per person, range 1–3). All participants gave informed consent. This study has approval from the University of Florida (IRB201901792) and University College London Research Ethics Committee (project 18839/001).

### Self-Perceptions of Aging

SPA were treated as a univariate latent variable. It was measured by five items from the Attitudes Toward Own Aging subscale of the Philadelphia Geriatric Center Morale Scale and another three items from the Berlin Aging Study to increase reliability as a univariate measure (J. Smith et al., 2017). Examples of the items include: “things keep getting worse as I get older,” “the older I get, the more useless I feel,” and “the older I get, the more I have to stop doing things that I like” (see Supplementary Table 1 for the full list). Each of these items was measured on a 6-point Likert scale, from strongly disagree to strongly agree. The original response scale was reverse coded, so that a higher value of the latent variable indicated more positive perceptions (Cronbach’s alpha = 0.81–0.83).

### Leisure Engagement

The LBQ included a 20-item Social Participation—Social Engagement scale, which included questions on a wide range of leisure activities (J. Smith et al., 2017). We excluded two items (childcare and watching television) that were not available in 2008, one item on adult care and another on using a computer as they were not considered leisure activities engaged in for pleasure during spare time. Further, we excluded religious participation (praying privately), focusing on secular participation only. This left us with 15 items which were then grouped into four domains based on exploratory factor analysis (Gao et al., 2023). The first domain was community activities including (1) volunteer work with children or young people, (2) other volunteer or charity work, (3) an educational or training course, (4) sport, social, or other club, and (5) meetings of nonreligious organizations. The second domain was cognitive activities: (6) reading, (7) word games, (8) play cards or games such as chess, and (9) writing. The third was creative activities: (10) home/car maintenance or gardening, (11) bake or cook something special, (12) make clothes, knit, embroider, etc., and (13) work on a hobby or project. The last domain was physical activities: (14) playing sports or exercising, and (15) walk for 20 min or more. These items were coded as 1 = daily, 2 = several times a week, 3 = once a week, 4 = several times a month, 5 = at least once a month, 6 = not in the last month/never/not relevant. All items were reverse coded, so a higher value indicated a higher level of leisure engagement. These items were summed and averaged to derive a univariate measure of leisure engagement, ranging from 0 to 6. Further, we derived four domain-specific measures (ranging from 0 to 6) to explore the possibility of differential associations with SPA.

### Covariates

We included a range of demographic, socioeconomic, and health-related covariates. Demographic variables were age groups (<60, 60–74, 75+ years), gender (men, women), partnership status (married/cohabitated, single/separated/widowed), and race/ethnicity (White [including Caucasian], Black [including African American], Other [including American Indian, Alaskan Native, Asian or Pacific Islander, Hispanic, Other]). In the public HRS data, this variable indicated the race/ethnicity as which participants primarily identified, and detailed information was removed to protect participant confidentiality. Socioeconomic covariates were educational attainment (none, high school, college, postgraduate), employment status (employed, not employed), total household income in quartiles (<$19,000, $19,000–$39,999, $40,000–$79,999, ≥$80,000). Finally, we included two health-related confounders: difficulties with activities of daily living and self-reported chronic health conditions (none, one, or more), indicating whether participants reported having diabetes, lung disease, cancer, heart conditions, high blood pressure, arthritis, complications from stroke, or other medical conditions. All covariates were measured at baseline.

### Statistical Analysis

Data were analyzed using the structural equation modeling (SEM) approach. SPA was treated as a latent variable in a reflective measurement model using confirmatory factor analysis (CFA, see Supplementary Figure 2). We tested longitudinal measurement invariance across the three waves by comparing the baseline CFA to metric and scalar invariance CFA models based on model fit indices (see Supplementary Table 2). As explained earlier, leisure engagement was treated as an observed variable measured by a formative model (VanderWeele, 2022).

**Figure 1.**
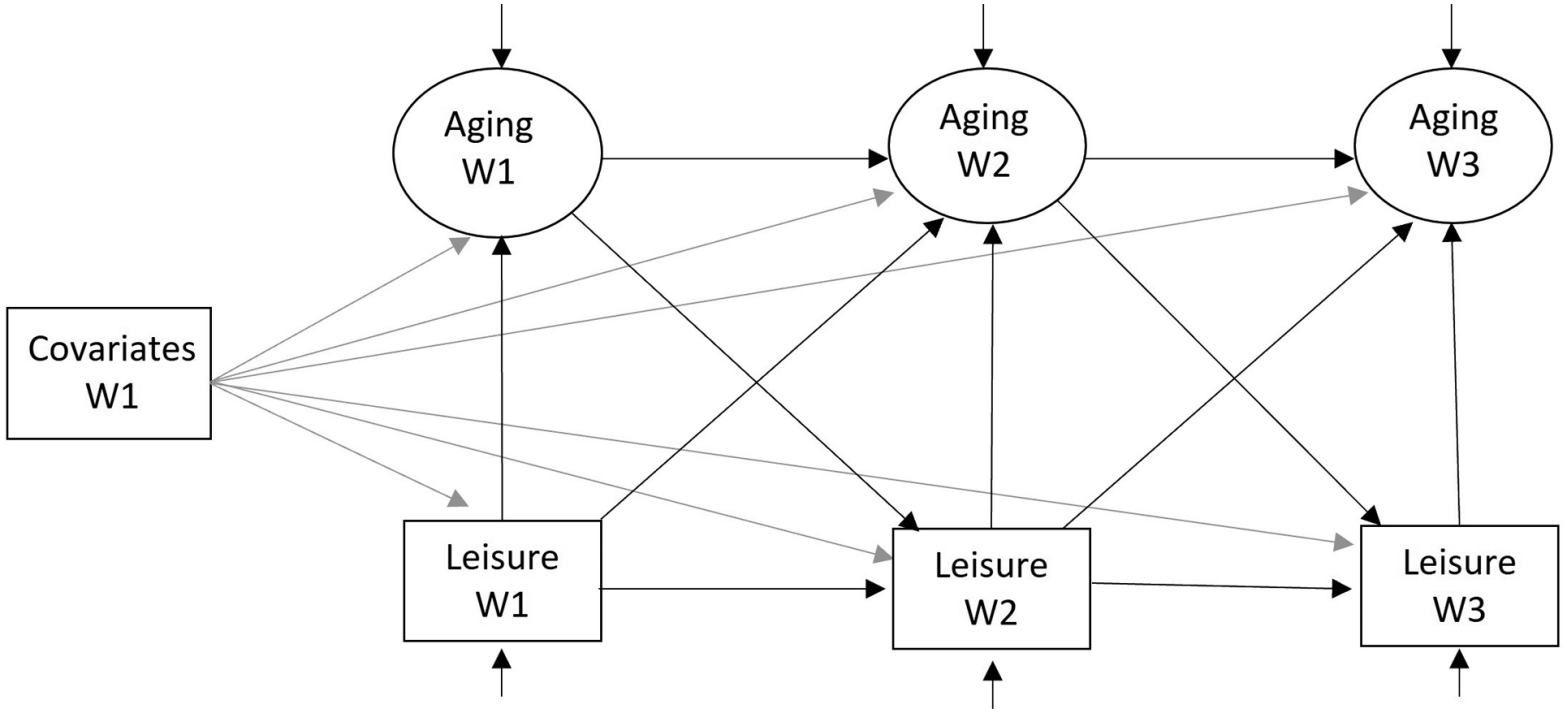
Model specification path diagram (omitting measurement models for simplicity).

Building on the measurement models where scalar measurement invariance across three waves was assumed, we fitted a path model including autoregressive paths for SPA and leisure engagement in each wave onto the same measure in the subsequent wave (Figure 1). Further, we tested both lagged and concurrent effects of leisure engagement on SPA. We also included a lagged effect of SPA on leisure engagement to examine the possibility of a reciprocal relationship. The model controlled for baseline covariates. The same model was fitted for each domain of leisure engagement instead of treating it as a univariate variable. A cross-lagged panel model with synchronous effect was chosen over the conventional cross-lagged panel model with synchronous correlations because the latter assumes that synchronous or concurrent effects between variables are zero. This is unlikely to be tenable given existing evidence and the 4-year interval between waves. Empirically, cross-lagged panel models with synchronous correlations led to poorer model fits (see Supplementary Table 3). Missing data on leisure engagement and SPA were handled using full-information maximum likelihood. Analyses were conducted in Mplus version 8.

## Results

In our analytical sample (*N* = 17,753), there were 34.6% of participants aged between 50 and 59, 58.0% women, 61.8% married or cohabitating, and 16.2% with less than high school education (Table 1). About 23.7% of participants self-identified primarily with a racial and/or ethnic background that is minoritized (Table 1). These numbers were largely in line with the U.S. national statistics in 2010 (U.S. Census Bureau, 2010).

**Table 1.** Sample Characteristics of the Analytical Sample (*N* = 17,753)

Variable	%
Age^a^	
<60	34.6
60–74	42.1
≥75	23.3
Gender	
Women	58.0
Men	42.0
Partnership status	
In partnership	61.8
Not in partnership	38.2
Ethnicity	
White	76.3
Black	16.1
Other	7.5
Education	
None	16.2
High school	53.0
College	20.7
Postgraduate	10.1
Employment	
Employed	37.9
Not employed	62.1
Household income	
<$19,000	20.8
$19,000–$39,999	25.3
$40,000–$79,999	26.9
≥$80,000	27.0
Chronic health conditions	
None	14.5
One or more	85.5
Number of ADL difficulties	
None	84.6
One or more	15.4

*Notes*: ADL = activities of daily living.

^a^Age ranging from 25 to 100, with a mean of 66 and standard deviation of 11.

**Figure 2.**
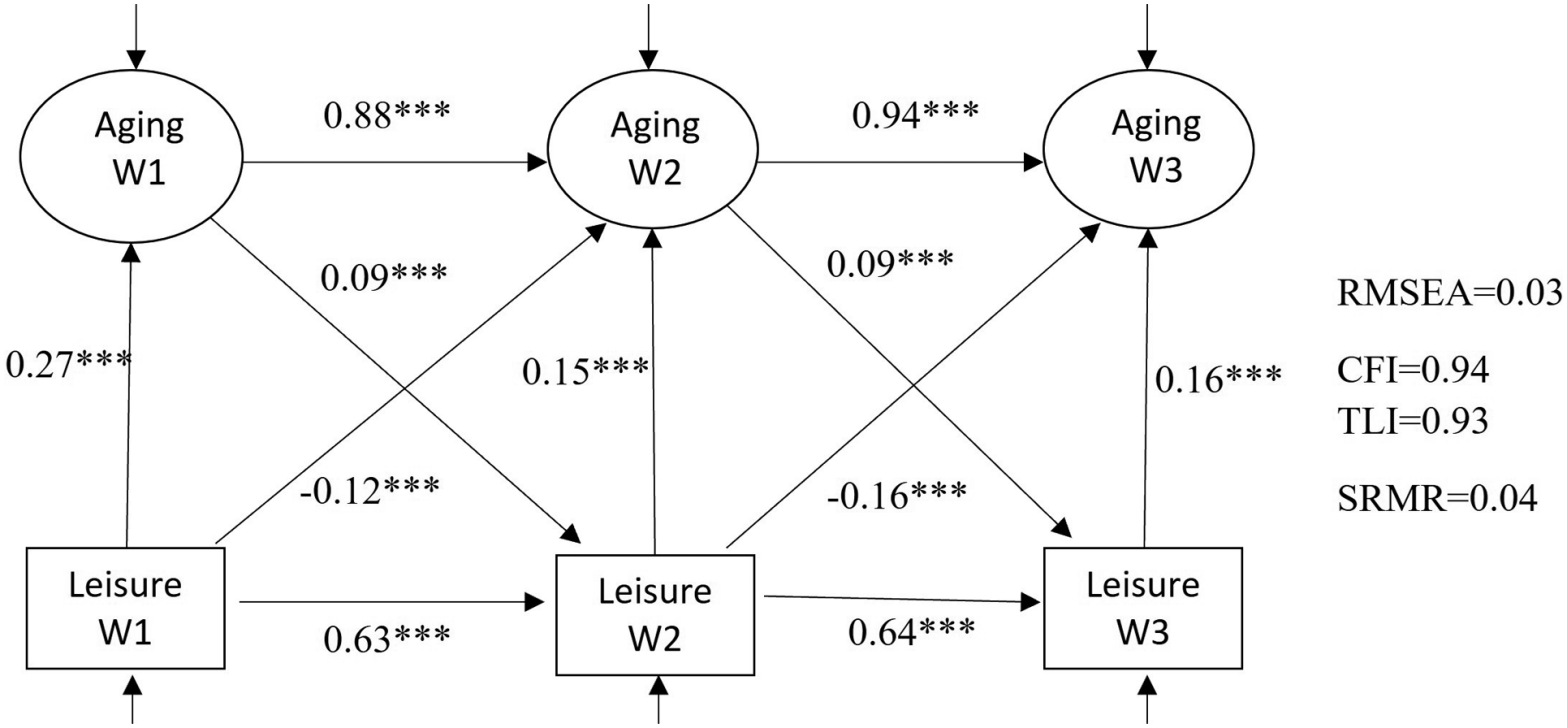
Standardized results from the full structural equation model using leisure engagement as a univariate measure. A higher value of the latent variable indicates a more positive perception of aging. CFI = comparative fit index; RMSEA = root-mean-square error of approximation; TLI = Tucker–Lewis index, SRMR = standardized root-mean-squared residual. ***p < .001; **p < .01; *p < .05.

Figure 2 shows the estimates of path analysis from the full SEM using leisure engagement as a univariate measure including all 15 items. The model fitted the data reasonably well (root-mean-square error of approximation [RMSEA] = 0.03, comparative fit index [CFI] = 0.94). Both SPA and leisure engagement significantly and positively predicted the same measures in subsequent waves (β = 0.63–0.94), showing stability over time. Concurrently, a higher level of leisure engagement was related to more positive SPA (β_w1_ = 0.27, *p*_w1_ < .001; β_w2_ = 0.15, *p*_w2_ < .001; β_w3_ = 0.16, *p*_w3_ < .001). The lagged leisure engagement was negatively associated with SPA (β_w1_ = −0.12, *p*_w1_ < .001; β_w2_ = −0.16, *p*_w2_ < .001). Given the same level of leisure engagement concurrently, a higher engagement in the previous wave (e.g., a higher decrease or lower increase in engagement between waves) was associated with less positive SPA in the current wave (Supplementary Figure 3). As hypothesized, there was also evidence of a reverse cross-lagged effect. More positive SPA in the previous wave were associated with a higher level of leisure engagement in the current wave (β_w1_ = 0.09, *p*_w1_ < .001; β_w2_ = 0.09, *p*_w2_ < .001). However, the effect sizes were relatively smaller in this model.

**Figure 3.**
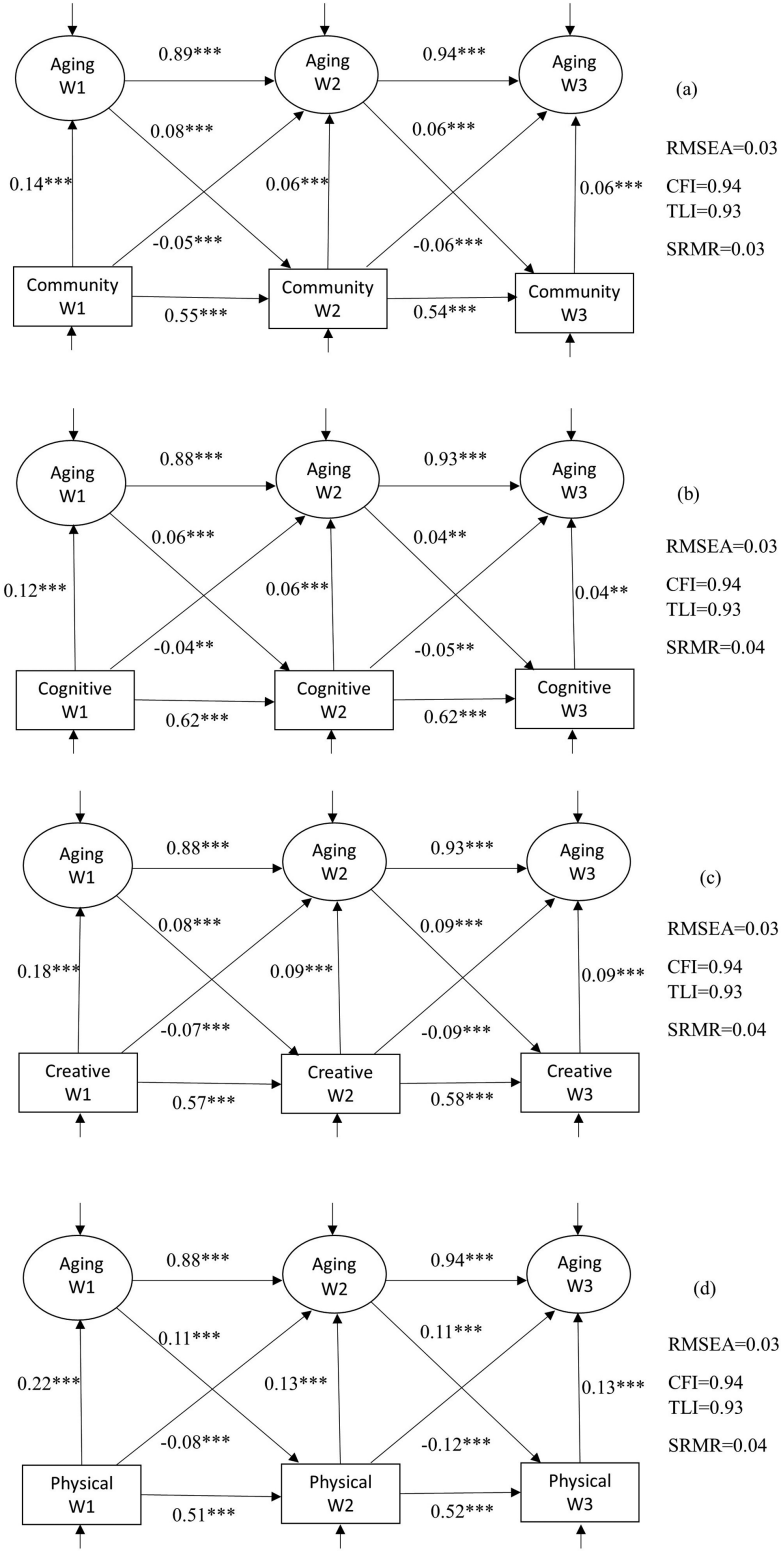
Standardized results from the full structural equation model on each domain of leisure engagement. A higher value of the latent variable indicates a more positive perception of aging. CFI = comparative fit index; RMSEA = root-mean-square error of approximation; TLI = Tucker–Lewis index, SRMR = standardized root-mean-squared residual. ***p < .001; **p < .01; *p < .05.

Further, we carried out analyses looking at each domain of leisure engagement separately, namely community activities, cognitive activities, creative activities, and physical activities. The results are presented in Figure 3. These models on specific domains also fitted the data well (RMSEA = 0.03, CFI = 0.94).

As shown in Figure 3A, a higher engagement in community activities was concurrently associated with more positive SPA (β_w1_ = 0.14, *p*_w1_ < .001; β_w2_ = 0.06, *p*_w2_ < .001; β_w3_ = 0.06, *p*_w3_ < .001). A higher level of engagement in community activities in the previous wave was associated with less positive SPA in the current wave, after controlling for concurrent engagement (β_w1_ = −0.05, *p*_w1_ < .001; β_w2_ = −0.06, *p*_w2_ < .001). Similarly, both concurrent (β_w1_ = 0.12, *p*_w1_ < 0.001; β_w2_ = 0.06, *p*_w2_ < 0.001; β_w3_ = 0.04, *p*_w3_ < 0.01) and lagged (β_w1_ = −0.04, *p*_w1_ < 0.01; β_w2_ = −0.05, *p*_w2_ < 0.01) effects of cognitive activities on SPA were found in the same direction as community activities (Figure 3B). Concurrent and lagged relationships with SPA were also found for creative activities (Figure 3C) and physical activities (Figure 3D). There was evidence supporting reciprocal relationships for all domains. For example, more positive SPA was associated with a higher level of engagement in physical activities in a subsequent wave (β_w1_ = 0.11, *p*_w1_ < .001; β_w2_ = 0.11, *p*_w2_ < .001). A higher level of engagement in physical activities in the previous wave was associated with less positive SPA in the current wave, after controlling for concurrent engagement (β_w1_ = −0.08, *p*_w1_ < .001; β_w2_ = −0.12, *p*_w2_ < .001). These findings were in line with the results from the overall model (Figure 2).

## Discussion

This study found consistent empirical evidence supporting our first hypothesis that leisure engagement predicts SPA. There was also evidence that SPA predicted leisure engagement (Hypothesis 2). Further, a reciprocal relationship of SPA with leisure engagement was found for all specific domains of leisure activities (Hypothesis 3).

We found consistent evidence for leisure engagement as a predictor of SPA, both concurrently and longitudinally, when using a univariate measure of leisure engagement and looking at specific domains (community, cognitive, creative, and physical activities). More specifically, a higher level of leisure engagement, regardless of the type of activities, was associated with more positive SPA among older adults. This is in line with previous literature on the benefits of leisure engagement among older adults (Adams et al., 2011; Noice et al., 2014; Stewart, 2005). The fact that all specific domains of leisure engagement were related to SPA suggests that there may not be any one unique “ingredient” of specific leisure activities that is responsible for the effects, but instead, the ability of leisure activities to activate multiple mechanisms of action related to aging perceptions may be key (Fancourt et al., 2021; Warran et al., 2022). This implies that older adults could choose any leisure activity based on their own interests and functional status, which would have similar benefits at least for their SPA. These findings provide empirical support for leisure as a resource for countering negative aging perceptions or stereotypes. Moreover, they shed light on the possibility of SPA as a mediator of the relationship between leisure engagement and health outcomes shown in the existing literature (e.g., Christensen et al., 2009; Levy et al., 2002).

Moreover, we found evidence for a reciprocal relationship where older adults’ SPA also predicted their leisure engagement, including the specific domains. This provides empirical evidence for the stereotype embodiment theory, in which older adults with a positive evaluation of their aging tend to have a more optimistic and resilient mindset, have a higher level of self-confidence, and are more likely to engage in health-promoting behaviors (Levy, 2009). This finding is consistent with previous research on specific leisure activities. For example, a longitudinal study of middle-aged and older adults showed that people with more positive SPA engaged in physical activity more frequently and were more likely to increase their physical activity over time (Wurm et al., 2008). It was also found that more negative SPA was associated with greater social disconnectedness (measured by social network size, social activities, volunteering, etc.) among older adults (Hu & Li, 2022). A study of community residents found that more positive SPA was associated with higher interest and more frequent participation in wellness programs (e.g., fitness classes, volunteer opportunities, social events, etc.; J. L. Smith et al., 2022). However, there is still a lack of empirical research on the impacts of SPA, and more research is needed to further understand the role of SPA on leisure engagement and other health behaviors.

In a recent paper, VanderWeele (2022) emphasized the importance of appropriate measurement for psychosocial constructs, in particular, the dimensionality of the exposure. Although we found all specific domains of leisure engagement predicted SPA (vice versa), these findings should not be taken as suggesting leisure engagement as a univariate construct or all leisure activities are equally beneficial. First, leisure is an umbrella term for a wide range of nonwork activities that people engage in during free time. Despite having data for 15 activities, there were other leisure activities (e.g., watching television, listening to music, shopping) that were not included in our analyses. Additionally, we only considered one type of classification for leisure activities (Gao et al., 2023). It is possible that a more exhaustive list of leisure activities may lead to a different classification and multidimensionality of leisure engagement. Second, these findings should be interpreted with respect to the study population and outcome measure. To better understand the dimensionality of leisure engagement and underlying causal mechanisms, empirical evidence is needed for other outcomes, age groups, and cultures. Third, although we examined the association of SPA with specific domains measured by conceptually related item sets, it is possible that these domains are not univariate themselves. Future research is encouraged to examine associations item by item (VanderWeele, 2022). In addition to the conventional reflective and formative measurement models, another possibility is that leisure activities all share different ingredients, overlapping to different degrees (Warran et al., 2022). Some activities may cluster together as sharing more ingredients than others, but that does not necessarily mean they are a fully defined category or dimension. Future studies could explore alternative conceptualization and measurement of leisure taking the ingredients perspective.

Our study has several strengths. HRS is a large nationally representative panel study, which allowed us to investigate both concurrent and longitudinal associations and examine the possibility of reciprocal relationships whilst controlling for a wide range of potential confounders. Due to the comprehensive measures of various leisure activities, we were able to look at different domains of leisure engagement. However, this study is not without limitations. There were only three waves of data wherein participants were followed up every 4 years. On the one hand, this prevented us from examining longitudinal associations within shorter follow-ups, which could influence the strength of the association. On the other hand, future research is needed to examine longitudinal changes of leisure engagement and SPA over a longer period when more waves of data become available. Moreover, additional research is also needed to better understand the nuances of gender and racial/ethnic identities and how they are related to leisure engagement and SPA, and to improve methods for measuring and accounting for structural racism in statistical research (Hardeman et al., 2022).

The SPA is an important indicator and promoter of healthy and successful aging. Our study has provided strong evidence for the potential benefits of leisure engagement on positive SPA, regardless of the type of activities. Moreover, we have shown that positive SPA could also increase the level of leisure engagement. SPA are, therefore, not only a product of leisure engagement, but also contribute to future engagement, potentially in a positive bidirectional cycle. For designing interventions, arguably it may be more effective to target leisure engagement, considering SPA are not only related to individual-level factors but also social–cultural contexts which take time to change. An increased leisure engagement may improve SPA and potentially enhance amenability to change, which comes with a wide range of other health benefits. Overall, our findings provide evidence for interventions and general guidelines for promoting leisure engagement among older adults.

## Supplementary Material

gbad182_suppl_Supplementary_Figures_S1-S3_Tables_S1-S3

## Data Availability

The raw HRS data are available from the RAND Center for the Study of Aging (https://hrsdata.isr.umich.edu/data-products/rand).
