# Supplementary material for: Leisure Engagement and Self-Perceptions of Aging: Longitudinal Analysis of Concurrent and Lagged Relationships
Source: J Gerontol B Psychol Sci Soc Sci. 2023 Dec 22;79(3):gbad182. doi: 10.1093/geronb/gbad182 (PMC10873860; doi:10.1093/geronb/gbad182)
Supplement: gbad182_suppl_Supplementary_Figures_S1-S3_Tables_S1-S3 [file gbad182_suppl_supplementary_figures_s1-s3_tables_s1-s3.docx]

**Online Supplementary Material**

Table S1 List of self-perceptions of aging questions

| Var name | Survey questions | Source |
| --- | --- | --- |
| Q29b1 | Things keep getting worse as I get older | ATOA^*^ |
| Q29b2 | I have as much as pep as I did last year | ATOA^*^ |
| Q29b3 | The older I get, the more useless I feel | ATOA^*^ |
| Q29b4 | I am as happy now as I was when I was younger | ATOA^*^ |
| Q29b5 | As I get older, things are better than I thought they would be | ATOA^*^ |
| Q29b6 | So far, I am satisfied with the way that I am aging | BAS^†^ |
| Q29b7 | The older I get, the more I have had to stop doing things that I liked | BAS^†^ |
| Q29b8 | Getting older has brought with it many things that I do not like | BAS^†^ |

Notes: ^*^ ATOA: Attitudes Toward Own Aging subscale of the Philadelphia Geriatric Center Morale Scale, BAS: Berlin Aging Study

Table S2 Comparing model fit indices across longitudinal factor analysis models on self-perceptions of aging

| Model | RMSEA | CFI | TLI | SRMR |
| --- | --- | --- | --- | --- |
| Configural invariance | 0.039 | 0.944 | 0.926 | 0.094 |
| Weak/metric measurement invariance | 0.037 | 0.944 | 0.934 | 0.094 |
| Strong/scalar measurement invariance | 0.036 | 0.943 | 0.937 | 0.094 |
| Strict/residual measurement invariance | 0.035 | 0.943 | 0.940 | 0.094 |

Notes: RMSEA=root mean square error of approximation, CFI=comparative fit index, TLI=Tucker–Lewis index, SRMR=standardized root mean square residual

Table S3 Comparing model fit indices between competing models

| Model | AIC | Δ_AIC_ | BIC | Δ_BIC_ |
| --- | --- | --- | --- | --- |
| **Overall leisure engagement** |  |  |  |  |
| CLPM-synchronous effects | 881,855 | -- | 882,782 | -- |
| CLPM-synchronous correlations | 1,218,960 | 337,105 | 1,219,948 | 337,166 |
| **Community activities** |  |  |  |  |
| CLPM-synchronous effects | 883,731 | -- | 884,658 | -- |
| CLPM-synchronous correlations | 1,218,302 | 334,571 | 1,219,291 | 334,633 |
| **Cognitive activities** |  |  |  |  |
| CLPM-synchronous effects | 896,928 | -- | 897,855 | -- |
| CLPM-synchronous correlations | 1,252,831 | 355,903 | 1,253,820 | 355,965 |
| **Creative activities** |  |  |  |  |
| CLPM-synchronous effects | 897,623 | -- | 898,550 | -- |
| CLPM-synchronous correlations | 1253696 | 356,073 | 1254685 | 356,135 |
| **Physical activities** |  |  |  |  |
| CLPM-synchronous effects | 910,132 | -- | 911,059 | -- |
| CLPM-synchronous correlations | 1,283,981 | 373,849 | 1,284,970 | 373,911 |

Notes: CLPM=cross-lagged panel model, AIC=Akaike Information Criterion, BIC=Bayesian information criterion

2016

2008

2010

2012

2014

2018

Wave 1 (baseline)

Wave 2

Wave 3

Figure S1 Panel data structure combining the two rotating random subsamples


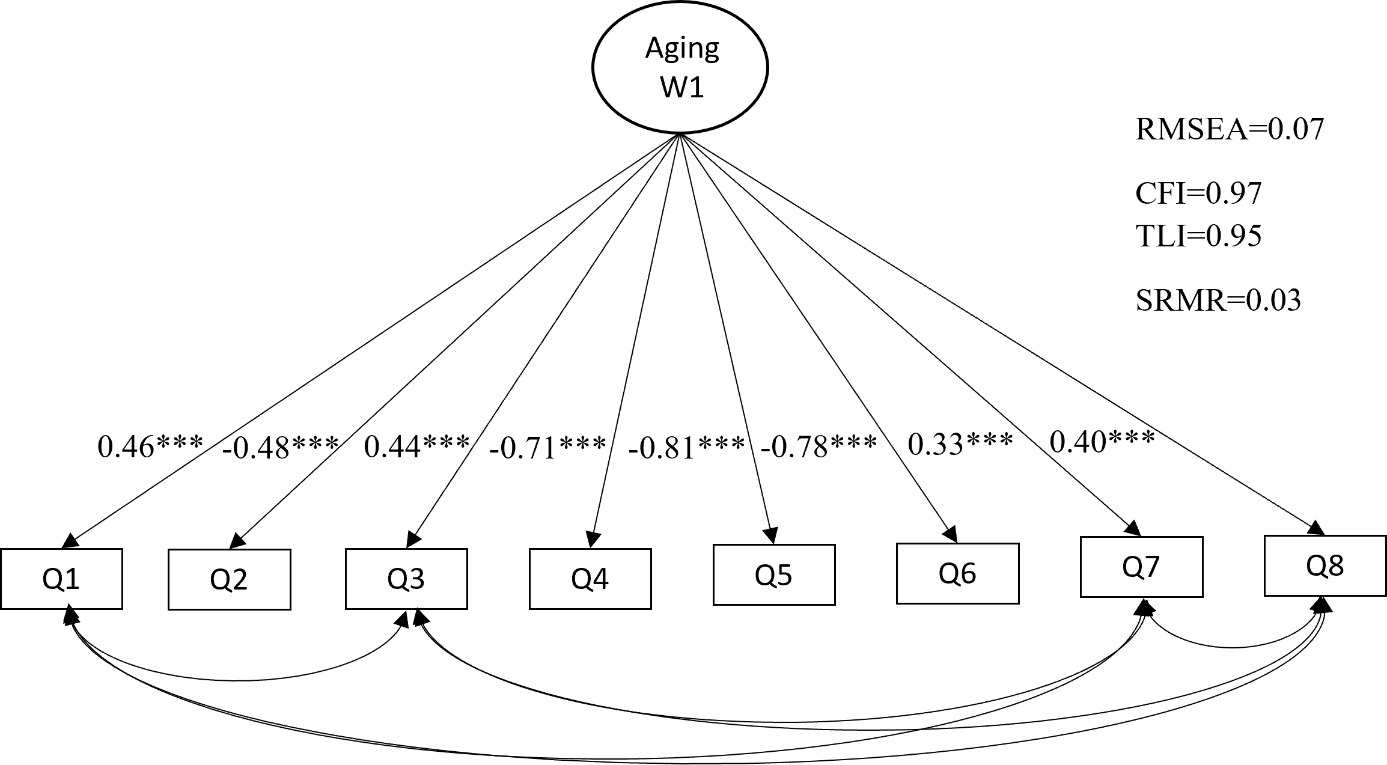


Figure S2 Measurement model of self-perceptions of aging at baseline wave (Q1-Q8 see Table S1, response scale: 1-strongly agree to 6-strongly disagree)


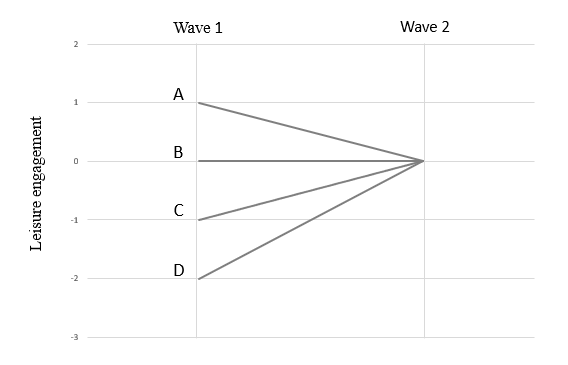


Figure S3 Hypothetical examples of leisure engagement changes between wave 1 and 2, holding wave 2 engagement the same
